# Game Customization and Pace Effects on Movement Performance and the User Experience During Serious Games for Balance Among People After a Stroke: Cross-Sectional Repeated Measures Study

**DOI:** 10.2196/88179

**Published:** 2026-06-17

**Authors:** Urška Puh, Nina Čelofiga, Judith E Deutsch

**Affiliations:** 1 Research and Development Center, University Rehabilitation Institute, Republic of Slovenia, Linhartova 51, Ljubljana, 1000, Slovenia, 386 1-4758-152; 2 Department of Physiotherapy, Faculty of Health Sciences, University of Ljubljana, Ljubljana, Slovenia, Slovenia; 3 Rivers Lab, Department of Rehabilitation and Movement Science, Rutgers School of Health Professions, Rutgers, The State University of New Jersey, Newark, NJ, United States

**Keywords:** active video games, exergaming, chronic stroke, neuromuscular intensity, movement performance, movement frequency, movement amplitude, user experience, balance, weight-shift training

## Abstract

**Background:**

Serious games can be custom or noncustom, each offering advantages for rehabilitation. Custom serious games developed specifically for rehabilitation allow control over feedback and adjustment of game speed and difficulty. Alternatively, noncustom games do not offer these controls but provide attractive graphics, sounds, and engaging game mechanics. The differences between these systems may affect users’ exercise intensity, quality of movement, and experience during gameplay, which have implications for system and game selection in rehabilitation.

**Objective:**

This study compared movement performance and user experience of patients after a stroke while playing custom and noncustom virtual reality balance games that were either game-paced or self-paced.

**Methods:**

A group of community-dwelling patients in the chronic phase post stroke participated in a cross-sectional repeated measures study. They were familiarized with 12 games; half were played using the custom system (Equio, Kinestica) and half with the noncustom system (Nintendo Wii Balance Board, Wii Fit games). Over 2 gameplay sessions with randomized blocks of game type (self-paced and game-paced) and the order of the system (custom and noncustom), participants played games requiring comparable movement of the center of pressure for each system. Movement performance (weight-shift repetitions and movement amplitude) was extracted from video recordings using the Kinovea software. User enjoyment (Modified Physical Activity Enjoyment Scale), flow (Flow State Scale for Occupational Tasks), and likability were assessed twice in each study block. Comparisons between the systems were conducted using paired *t* tests or Wilcoxon tests. Effect sizes (ES) were calculated using Cohen *d*.

**Results:**

Twenty-six participants (aged 27‐70 years) were included in the analysis. Repetitions and movement amplitude were significantly greater (repetitions: *P*˂.001–.003; amplitude: *P*=.002–.03) with large ES for all noncustom game–paced games and for half of the noncustom self-paced games (repetitions: ES 0.8‐2.5; amplitude: ES 0.6‐0.9). Perception of exertion was greater for noncustom game-paced games (ES 0.6; *P*=.005). In contrast, users’ flow state (total and subdomains of sense of control and emotional experience; ES 0.6‐0.8; *P*≤.03) and user preference were significantly greater for custom game-paced games. There was no significant difference between custom and noncustom games for enjoyment.

**Conclusions:**

Serious gameplay was influenced by customization and type of game pacing, both of which should be considered when selecting serious games for balance training. Repetitions, movement amplitude, and perception of effort were greatest for the noncustom game-paced games, indicating greater intensity, while also addressing the difficulty patients after a stroke have with weight-shifting to the affected side. In contrast, custom games provided a sense of control and increased flow state, which may promote better adherence. Findings from this study have implications for clinicians and game designers. Both custom and noncustom games offer training features that support stroke rehabilitation.

## Introduction

For patients who are ambulatory after a stroke, virtual reality (VR)–based balance exercises and serious games, when combined with conventional therapy, have been shown to be more effective in improving balance than conventional therapy alone [1]. This is true for patients who are less than [2] and more than 6 months [3,4] after a stroke. Additionally, for patients in the chronic phase post stroke, VR balance training alone or combined with other therapies is also more effective than comparable treatments for improving mobility [3-5]. Recent meta-analyses [5,6] have confirmed that VR, compared to alternative interventions or as an adjunct to usual care, may be beneficial for improving balance and may probably reduce activity limitations after a stroke. However, due to substantial heterogeneity and risk of bias across studies, the findings regarding the magnitude and clinical relevance of effects related to dynamic balance should be interpreted with caution [5]. Systematic reviews [2,5] and clinical practice guidelines [7,8] recommend combining balance training with VR or serious games for patients after a stroke. Specifically, VR balance training in stance can be performed with pressure-sensitive balance boards that provide information about the movement of the user’s center of pressure (COP) for weight-shift training [7].

VR technologies delivered as serious games may enhance balance rehabilitation in patients after a stroke by incorporating engagement, feedback, repetition, cardiovascular and neuromuscular exercise intensity, and task-oriented training [7]. Interventions using VR can be effective because of their ability to mobilize recovery mechanisms and support physiotherapy goals [9]. For example, they promote movement frequency and movement amplitude toward the affected side, which are markers for increased use of the paretic limb and achieving the therapeutic dose required for neuroplasticity and functional recovery [10]. Enjoyment of serious games and the flow state support motivation and engagement, which are necessary for therapy adherence and duration to derive benefits such as motor learning and improved functioning [11,12].

Noncustom games, such as the Nintendo Wii, originally created for entertainment or recreation, can be adapted for rehabilitation, while custom games are specifically designed for rehabilitation. The Nintendo Wii console, along with the Wii Balance Board and Wii Fit games, is the most clinically accessed noncustom serious game system [13,14], and these games are used most often in poststroke rehabilitation [15]. Custom systems are designed to incorporate neurorehabilitation and motor learning principles for movement recovery, making them potentially more effective than noncustom systems [9]. Custom systems allow for the adjustment of game speed and control of game mechanics (eg, amplitude, feedback, and dual tasking). In contrast, noncustom games are more complex and less adaptable to the needs of individual patients [16], but they generally provide attractive graphics and sounds.

The nature and application of serious games in rehabilitation after a stroke are still not fully understood. An important critique of noncustom systems for rehabilitation is that they do not facilitate desired movement patterns and therefore do not achieve therapeutic goals [16-19]. A study of patients in the chronic phase post stroke [20] showed that using noncustom VR games resulted in 5 times more repetitions and double the acceleration of affected upper limb movements compared to traditional therapy, but 3 months of training did not improve motor function in any group. It has also been shown that patients can engage with noncustom technology during rehabilitation when a therapist tailors the prescription [21]. However, the perceived benefit of using technology, along with receiving sufficient support to enable its use, appears to influence the level of patient engagement [21].

Both custom and noncustom games can be further divided into two game types: (1) self-paced games, in which speed is controlled by the user; and (2) game-paced games, in which speed is set by the game. Game pace is an important feature that has not been well-studied and is often cited as a limitation for patients after a stroke to engage with games [19].

While efficacy differences between custom and noncustom systems for upper limb training have been documented in one meta-analysis [22], and no difference in benefits was found in another meta-analysis [6], the differences for balance training post stroke are not yet known. Comparing custom and noncustom VR games for balance in the same group of patients after a stroke can provide insights into how game customization and type of game pacing influence gameplay. We have directly compared custom and noncustom game-paced VR stepping games for patients after a stroke and found that the custom game provided lower movement frequency (number of stepping repetitions) but better step accuracy, greater enjoyment and likability, and lower perceived exertion than the noncustom game [23].

In this study, we sought to extend the findings on game customization to include the type of game pacing. Therefore, the purpose of this study was to compare how patients after a stroke played custom and noncustom VR balance games that were either game-paced or self-paced and to investigate how movement performance (measured by movement frequency and movement amplitude) and user experience (exertion, enjoyment, flow, and preferences) differed. We hypothesized that patients after a stroke would have greater movement frequency (number of weight-shift repetitions to the affected lower limb) during noncustom games. In contrast, we hypothesized that users would have greater pelvic displacement to the affected side and report a better experience with custom game-paced games, but there would be no difference in user experience with self-paced games.

## Methods

This study used a single-group, cross-sectional, repeated measures design and was registered at ClinicalTrials.gov (NCT06463730).

### Participants

**Figure 1.**
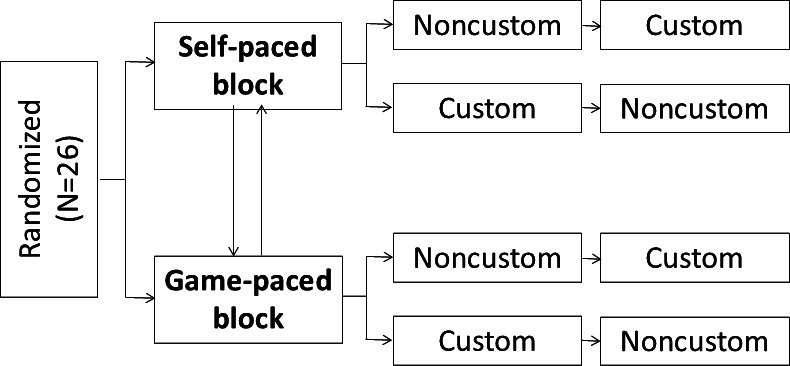
Flow diagram of the study participants: All participants completed both study blocks in a randomized order of game types (self-paced and game-paced) and systems’ order (custom and noncustom). Only one block was played per session.

A convenience sample of community-dwelling patients was recruited through members of the Association of Cerebrovascular Disease Patients. Adults in the chronic phase post stroke (>6 mo) were eligible to participate if they had a stable health condition, could follow simple instructions, and were able to walk with supervision or independently, with or without a walking aid, on flat ground or all surfaces (Functional Ambulation Categories [FAC] 3‐5) [24]. Participants were excluded if they could not play games on the balance boards or had additional neurologic or musculoskeletal impairments that would interfere with performing the required tasks or completing the questionnaires. Participants were block randomized into 4 combinations (Figure 1).

### Ethical Considerations

The study complied with the Declaration of Helsinki and was approved by the National Medical Ethics Committee of the Republic of Slovenia (0120-303/2021/3). All participants provided written informed consent to participate in the study. All data were anonymized. Participants did not receive any compensation.

### Description of Custom and Noncustom Systems and Games

**Figure 2.**
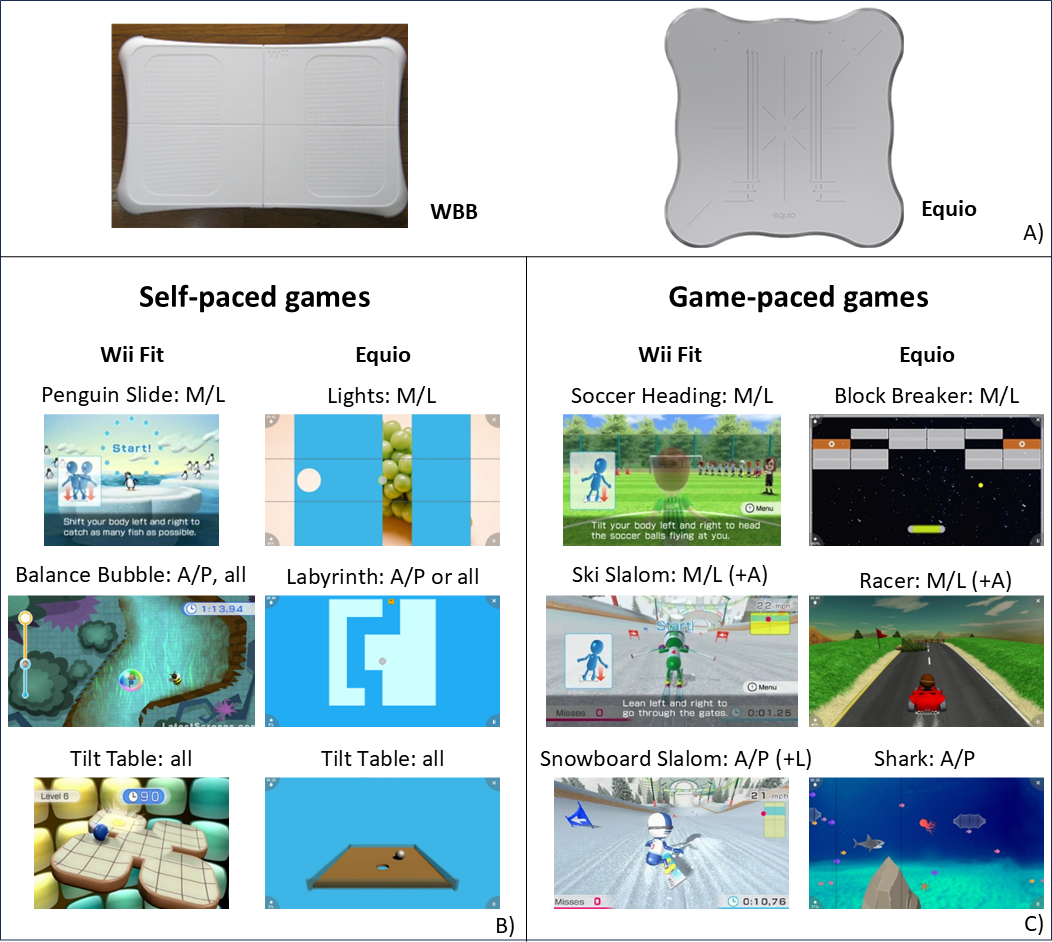
(A) The noncustom system Nintendo Wii Balance Board (WBB) and the custom system (Equio) with their (B) self-paced games and (C) game-paced games, and the directions of center of pressure movement. +A: anterior; A/P: anteroposterior; +L: lateral; M/L: mediolateral.

The custom system, classified as a class 1 medical device and developed for rehabilitation, was the Equio (Kinestica, d.o.o.). The noncustom system was the Nintendo Wii with the Wii Balance Board and Wii Fit games. These systems were chosen because they allow comparisons in game mechanics, as both use a balance board to interface with the game. The balance boards of both systems have 4 pressure sensors at the bottom, which calculate the COP position of the user based on body-weight transfers during standing, and both are portable. The custom board has a sampling frequency of 200 Hz [25], while the noncustom board has a sampling frequency between 35 and 100 Hz (average 63 Hz) [26]. The custom board is made of aluminum, is thinner, and has a larger surface area (496 mm × 496 mm × 12 mm; 6635 g) [25], while the noncustom board is made of plastic, is lighter, but thicker (510 mm × 310 mm × 55 mm; 4000 g; Figure 2A). The systems were connected to a TV screen 220 cm away from the participant standing on the balance boards.

Comparable self-paced and game-paced games from each system were selected based on the required movements of the center of COP direction (see Figure 2B and C). For example, the tilt table game, present in both systems, required the COP to be moved in all directions. For the noncustom games, the basic difficulty level was used for all participants, as recommended by the developer for initial gameplay. The custom games have 3 difficulty levels. Their difficulty was adjusted for accuracy requirements (eg, size of obstacles or targets), number of targets, and the addition of a dual task. Game-paced games were played at the speed required by the game, while self-paced games were played at the participants’ preferred speed. Details of custom and noncustom games are described in Multimedia Appendix 1.

### Protocol

**Figure 3.**
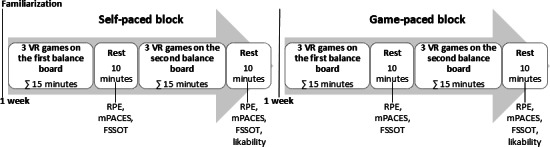
The study protocol: familiarization and 2 gameplay sessions (the order of the study conditions was randomized), including assessment with the Flow State Scale for Occupational Tasks (FSSOT), the Modified Physical Activity Enjoyment Scale (mPACES), the Borg Perceived Exertion Scale (RPE), and likability-user preference. VR: virtual reality.

Participants attended a familiarization session and 2 gameplay sessions, approximately 1 week apart, in the Physiotherapy Laboratory of the university (see Figure 3). Familiarization consisted of playing each of the 12 games for 2 minutes. To set the range of motion needed during the custom games, the COP range of motion in the mediolateral and anteroposterior directions was measured for each participant using the custom system. These values were automatically translated to the custom game scenarios and adjusted based on accuracy at the start of gameplay in all sessions. The difficulty level of each custom game was determined based on the capabilities of participants during the familiarization session.

Gameplay was studied in 2 sessions. To ensure movement quality and safety, during the first minute of each game, a physiotherapist guided movement with either verbal or manual cues and positioned the participant’s feet. Both the order of game types (self-paced and game-paced) and the order of the systems (custom and noncustom) were randomized (see Figure 1). In each gameplay session, participants played 1 block consisting of 3 games (5 min each) on the noncustom balance board and 3 comparable games on the custom balance board. Each gameplay session consisted of 30 minutes of training with a 10-minute rest in the middle. During the rest periods, the assessment of the user’s experience was performed in a sitting position (Figure 3).

### Data Collection and Reduction

Participants’ balance and gait were measured during the familiarization session. Balance was assessed using the Mini Balance Evaluation System Test [27,28]. Walking capacity was rated with the FAC, and comfortable and fast walking speeds were measured with the 10- meter walk test. The latter was conducted on a 14-meter walking path [28], with 1 familiarization trial and 1 test trial performed at a comfortable speed, followed by a fast speed walking trial.

**Figure 4.**
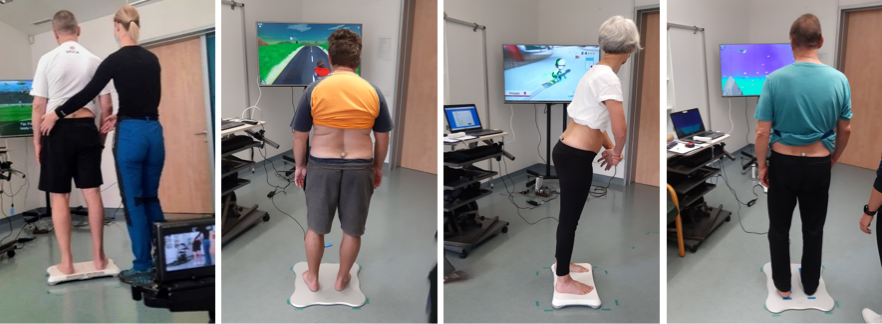
Participants after a stroke playing games with the noncustom system (first and third) and the custom system (second and fourth) balance boards.

The primary outcome measures were movement frequency (number of weight-shift repetitions to the affected lower limb) and movement amplitude of pelvic displacement to the affected side. Movement performance during gameplay was recorded using a digital video camera (Canon PowerShot SX620 HS; Figure 4, first), recording at 30 frames per second. A marker used to monitor pelvic movement was placed on the sacrum at the height of the center of body mass (S2). The camera was positioned behind the participant to clearly view the participant’s pelvis and torso, the marker, the balance board, the position of the feet, and the TV screen. The TV screen, balance boards, and camera were always positioned in the same place; however, the camera was moved back from this position if the participant’s head was not visible in the video. The first minute of the recordings was excluded from the analysis because participants had to adjust to the game at the beginning (described above).

**Figure 5.**
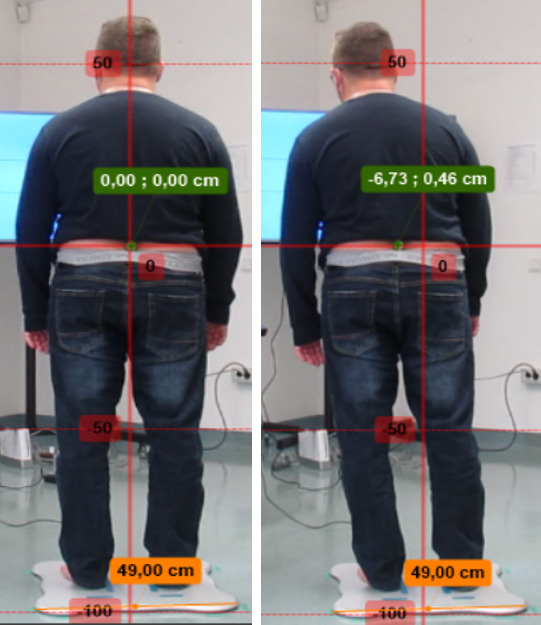
Analysis of movement performance during gameplay on video recordings using the Kinovea software. The center of the coordinate system and the green dot/marker were overlaid at the start. The first number (x-coordinate) represents the position of the pelvic marker (0.00 cm) at the start and its mediolateral displacement at the end position (−6.73 cm), indicating a weight shift toward the left (stroke-affected) leg in the frontal plane. The orange reference indicates the length of the balance board, which was used to calibrate distance measurements.

For the 8 games that required movement of the COP in either the mediolateral direction or all directions, the number of weight shifts to the affected lower limb (repetitions/min) and the amplitude of pelvic displacement to the affected side in the frontal plane were extracted from video recordings using the Kinovea software (version 0.9.1) [29]. For each game, the last 4 minutes of gameplay were analyzed. Any voluntary movement of the pelvis to the affected side in the mediolateral direction, from the starting point to the farthest point and back to the starting position, was considered a correct repetition. The amplitude of pelvic displacement was determined by measuring the maximum displacement of the marker from the start to the end position (Figure 5). The analysis was performed for games in which the markers were not concealed in ≥50% of the recording (eg, due to compensatory rotation of the pelvis or while the physiotherapist provided additional guidance or safety).

The secondary outcome measures assessed user experience: the Borg Rating of Perceived Exertion Scale (RPE) [30], the Modified Physical Activity Enjoyment Scale (mPACES) [31], and the Flow State Scale for Occupational Tasks (FSSOT) [32]. These measures were collected twice in each study block, immediately after playing the games. The Borg Scale (ranging from 6 to 20) was used to estimate the cardiovascular intensity of physical activity through RPE. The mPACES is a 5-item scale, with scores expressed as percentages, that rates enjoyment during gameplay and has been used previously in VR studies [23,31]. The FSSOT is a 14-item questionnaire assessing flow state, with scores expressed as a total or in 3 domains: sense of control, emotional experience, and absorption [32]. At the end of each gameplay session, participants viewed a figure displaying the 6 games and ranked them from most liked (6 points) to least liked (1 point).

### Data Analysis

Data were assessed for normality using the Shapiro-Wilk test. Paired *t* tests with CIs were used to determine differences between custom and noncustom games for the number of repetitions and amplitude of pelvic displacement within the game pairs. Missing data were excluded from the analysis. Paired *t* tests or Wilcoxon signed rank tests were conducted to test for differences in mPACES, FSSOT, and RPE scores between games of custom and noncustom systems within self-paced and game-paced games. Cohen *d* was calculated to estimate the ES (0.2 small; 0.5 medium; >0.8 large). Cohen *d* and CIs for nonparametric data were estimated using the Bootstrap method. To correct for multiple comparisons, the α was adjusted to .025 within pairs. To identify differences in likability within self-paced and game-paced games, the total score of each game was calculated for all participants and then ranked within each block from most liked (highest total score) to least liked (lowest total score). IBM SPSS Statistics version 29.0 was used for the analysis.

## Results

### Participants

Twenty-six participants completed the study. Two individuals were excluded from the original 28 because they were unable to play the games on the balance board. There were no adverse events (eg, falls) or side effects (eg, motion sickness). Some movement performance data were missing due to concealed pelvic marker.

Participants’ ages ranged from 27 to 70 years. The time since experiencing a stroke ranged from 0.7 to 37.8 years. Most participants were able to walk independently on both level and nonlevel surfaces, stairs, and inclines (FAC 5; Table 1). Participants’ balance ranged from moderate (8‐13 points; n=4) to mild balance deficits (15‐23 points; n=14) and very mild balance deficits to normal balance (24‐27 points; n=8) [33].

**Table 1.** Participants’ characteristics: demographics, clinical characteristics, and balance and walking abilities (N=26).

Characteristic	Value
Age (y), mean (SD)	56.0 (10.0)
Sex, n	
Male	16
Female	10
Time since stroke (y), mean (SD)	11.6 (10.4)
Lesion type, n	
Ischemic	10
Hemorrhagic	9
Both	4
Unknown	3
Stroke affected side, n	
Left	11
Right	13
Bilateral	2
FAC^a^ (n)	
3	2
4	6
5	18
Walking aid in the community, n	
None	14
Walking stick	9
Crutch	1
Rollator	2
Ankle-foot orthosis (n)	2
Mini-BESTest^b^ (score: max 28), mean (SD)	20 (5.5)
10MWT^c^_comfortable (m/s), mean (SD)	1.00 (0.33)
10MWT_fast (m/s), mean (SD)	1.38 (0.47)

a FAC: Functional Ambulation Categories.

b Mini-BESTest: Mini Balance Evaluation Systems Test.

c 10MWT: 10-meter Walk Test.

### Movement Performance

#### Movement Frequency

For self-paced games, the movement frequency (number of weight-shift repetitions to the affected lower limb) was significantly higher for the noncustom game Penguin Slide compared to the custom game requiring comparable mediolateral movement. There were no significant differences between the 2 Tilt Table games (Table 2). For game-paced games, movement frequency was significantly higher for both noncustom games (Soccer Heading and Ski Slalom) than for their custom paired games (Table 2).

**Table 2.** Movement performance: comparisons of movement frequency (number of weight shifts to the affected lower limb) and movement amplitude (pelvic displacement to the affected side) within game pairs of the custom and noncustom systems^a^.

System	Game	Mean (SD)	*t*-test			ES^b^
			95% CI	*t* (*df*)	*P* value	
Movement frequency (reps/min)						
Self-paced						
Noncustom	Penguin Slide	10.8 (2.1)	4.6 to 6.8	10.69 (18)	<.001^c^	2.5
Custom	Lights	5.1 (0.7)				
Noncustom	Tilt Table	7.1 (2.0)	−0.2 to 1.5	1.59 (16)	.13	0.4
Custom	Tilt Table	6.5 (1.3)				
Game-paced						
Noncustom	Soccer Heading	12.4 (2.9)	6.0 to 8.9	10.77 (17)	<.001^c^	2.5
Custom	Block Breaker	5.0 (1.5)				
Noncustom	Ski Slalom	9.0 (1.9)	0.7 to 3.1	3.44 (18)	.003^c^	0.8
Custom	Racer	7.1 (2.0)				
Movement amplitude (cm)						
Self-paced						
Noncustom	Penguin Slide	9.2 (3.2)	−0.5 to 3.1	1.55 (17)	.14	0.4
Custom	Lights	7.9 (2.8)				
Noncustom	Tilt Table	8.4 (2.2)	0.2 to 2.0	2.48 (16)	.025^c^	0.6
Custom	Tilt Table	7.4 (2.2)				
Game-paced						
Noncustom	Soccer Heading	10.5 (3.3)	0.8 to 3.1	3.65 (15)	.002^c^	0.9
Custom	Block Breaker	8.5 (3.5)				
Noncustom	Ski Slalom	9.3 (3.0)	0.2 to 2.5	2.56 (13)	.024^c^	0.7
Custom	Racer	7.9 (2.9)				

a Games requiring center of pressure (COP) movement only in anteroposterior directions were excluded from this analysis.

b ES: effect size of the difference.

c Statistically significant difference for comparison between the 2 conditions with post hoc correction for multiple comparisons *P*≤.025.

#### Movement Amplitude

For self-paced games, the amplitude of pelvic displacement to the affected side was significantly greater for the noncustom game Tilt Table than for the custom one. There were no significant differences within the mediolateral direction game pair (Table 2). For game-paced games, the amplitude of pelvic movement was significantly greater for both noncustom games than for their custom-paired games (Table 2).

### User Experience

#### Enjoyment

For both self-paced and game-paced games, there was no statistically significant difference in mPACES scores between custom and noncustom games. Notably, for game-paced games, mPACES scores were on average 8 points higher for custom games and showed a moderate ES (Table 3).

**Table 3.** User experience: ratings of enjoyment of physical activity, flow state, and ratings of perceived exertion between the custom and noncustom systems’ games within self-paced and game-paced game types (N=26).

Game type and system	Mean (SD)	Wilcoxon test or *t* test			ES^a^
		95% CI	*Z*/*t* (*df*)	*P* value	
mPACES^b^ %					
Self-paced					
Noncustom	81.2 (13.6)	−3.9 to 6.5	−0.52	.60	0.1
Custom	79.9 (16.4)				
Game-paced					
Noncustom	73.0 (16.5)	−15.8 to 1.6	−2.09	.04	−0.5
Custom	81.2 (14.0)				
FSSOT^c^					
Sense of control					
Self-paced					
Noncustom	31.8 (7.0)	−4.6 to 1.0	−1.11	.27	−0.3
Custom	33.7 (5.7)				
Game-paced					
Noncustom	30.0 (6.2)	−6.2 to 2.2	−3.33	<.001^d^	0.8
Custom	34.2 (5.1)				
Emotional experience					
Self-paced					
Noncustom	23.3 (4.9)	−1.0 to 1.5	−0.44	.66	0.1
Custom	23.0 (4.8)				
Game-paced					
Noncustom	22.6 (4.4)	−2.8 to 0.7	−2.50	.01^d^	−0.6
Custom	24.3 (4.5)				
Absorption					
Self-paced					
Noncustom	22.8 (3.9)	−1.2 to 0.8	−0.16	.88	−0.1
Custom	23.0 (3.8)				
Game-paced					
Noncustom	21.6 (4.3)	−3.0 to 0.2	−2.22	.03	−0.4
Custom	23.2 (4.0)				
Total score					
Self-paced					
Noncustom	77.8 (14.0)	−6.1 to 2.5	−0.54	.59	−0.2
Custom	79.7 (12.8)				
Game-paced					
Noncustom	74.2 (12.9)	−11.4 to 3.7	−3.15	.002^d^	−0.7
Custom	81.7 (12.7)				
RPE^e^					
Self-paced					
Noncustom	12.2 (2.3)	−1.1 to 0.4	−0.95 (25)^f^	.35	0.2
Custom	12.5 (1.9)				
Game-paced					
Noncustom	12.4 (2.5)	0.4 to 2.0	3.04 (25)^f^	.005^d^	0.6
Custom	11.2 (2.2)				

a ES: effect size of the difference.

b mPACES: Modified Physical Activity Enjoyment Scale.

c FSSOT: Flow State Scale for Occupational Tasks.

d Statistically significant difference for comparison between the 2 conditions with post hoc correction for multiple comparisons *P*≤.025.

e RPE: Borg Rating Scale of Perceived Exertion.

f *t* test values (df).

#### Flow State

For self-paced games, there were no significant differences in FSSOT scores between custom and noncustom games. For game-paced games, the total FSSOT score was on average 8 points higher for custom games than for noncustom games. This difference was statistically significant for the total FSSOT score and for its subscores—sense of control and emotional experience (Table 3).

#### Perceived Exertion

The RPE scores ranged from 7 to 17. For the self-paced games, there were no significant differences in RPE between the custom and noncustom games. For the game-paced games, RPE was significantly greater for the noncustom games; however, both mean scores fell within the light-to-moderate range of perceived exertion (Table 3).

#### Likability—User Preference

For self-paced games, participants liked the Tilt Table of the custom system best, followed by Lights (custom system), and then the 3 noncustom games: Balance Bubble, Penguin Slide, and Tilt Table. The least liked game was Labyrinth (custom system; Table 4). For game-paced games, the 3 custom games were preferred. The noncustom games received the fewest points, especially Soccer Heading and Snowboard Slalom (Table 5).

**Table 4.** Likability of the custom and noncustom systems’ games within the self-paced game type (N=26).

Likability	Noncustom			Custom		
	Penguin	Balance Bubble	Tilt Table	Lights	Labyrinth	Tilt Table
Sum	93	95	90	98	57	113
Mode	3	3	1	6	1	5
Median (IQR)	3 (2.7-5.0)	3.5 (2.8-5.2)	4 (1.0-5.0)	4 (2.0-5.3)	1.5 (1.0-3.2)	5 (3.0-6.0)

**Table 5.** Likability of the custom and noncustom systems’ games within the game-paced game type (N=26).

Likability	Noncustom			Custom		
	Soccer Heading	Ski Slalom	Snowboard	Block Breaker	Racer	Shark
Sum	65	91	58	94	125	113
Mode	2	2	1	4	6	5
Median (IQR)	2 (1.0-3.0)	3 (2.0-5.0)	2 (1.0-3.2)	4 (2.0-5.0)	5 (3.8-6.0)	5 (3.7-5.0)

## Discussion

### Principal Results

Participants demonstrated better movement performance, including higher movement frequency (more weight-shift repetitions) and greater pelvic displacement to the affected side during the game-paced noncustom games. Movement performance between custom and noncustom games was more similar during self-paced games. User experience, specifically flow state and preference, favored the game-paced custom games.

As hypothesized, movement frequency based on the number of weight shifts to the affected lower limb during custom games was on average 21% to 61% lower than during noncustom games, with large ESs (ES 0.8‐2.5). These findings are consistent with our previous study [23], where the total number of step repetitions was significantly lower for the custom game compared to the noncustom Kinect game (average difference 33%). The custom system allowed calibration for movement displacement within the user’s limits of support as well as selection of movement speed (game difficulty level). In contrast, noncustom games lack this customization, requiring players to move faster and farther to meet the game mechanics. This could, however, result in poor movement control, as shown in our previous work [23].

Contrary to our hypothesis, the amplitude of pelvic displacement toward the affected side was on average 13% to 21% greater for the noncustom games than for the custom games, with medium-to-large ESs (ES 0.6‐0.9), except for one case (self-paced games: Penguin Slide vs Lights), where there was no difference. This may be due to a conservative range of motion settings for custom games, which could only be adjusted in 5 centimeter increments. The movement boundary set by customization likely limited potential displacements. However, without a 3D motion analysis, no definitive statement can be made about movement quality or lack of control in relation to movement amplitude.

As hypothesized, user experience, specifically flow state, was better for the custom game-paced games than for the noncustom equivalent games, and there was no difference between custom and noncustom self-paced games. These findings support the claim that the lack of user control is especially evident in game-paced, rather than self-paced, games [23]. An important finding is that the flow state (FSSOT) data deepen the understanding of the user experience during custom game-paced games, in which subscores for sense of control and emotional experience were significantly higher than for noncustom games. This suggests that, regardless of the serious game system used, the ability of the user to control game speed is an important feature.

The findings of flow-state–favoring, custom game–paced games in this study were supported by a medium ES (ES 0.5) in enjoyment. The construct of enjoyment has been reported to be related to engagement for training and motor learning in virtual environments and likely promotes motivation [12]. VR-game balance training, specifically, was reported to be more enjoyable than conventional weight-shift training [34]. Therefore, it is important to consider the type of game pacing as a factor for the usability of serious games for patients with neurological conditions. Game-paced games use speed to determine the level of difficulty, which, when not adjusted, can lead to frustration and affect game enjoyment and motivation [19].

There was a small but statistically significant difference in perceived exertion, with lower RPE scores for the custom games, indicating a medium ES (ES 0.6). Participants perceived the game-paced noncustom games as somewhat more exerting (a mean difference of 1.19 points) than the custom games. This confirms previous findings on perceived exertion [23] and is consistent with the greater movement frequency and pelvic displacement observed in this study. However, perceived effort was quite similar across the games, and the difference was not clinically meaningful. The range of RPE scores for the game-paced noncustom games was wide, from 7 (very, very light) to 17 (very hard), and similar for the custom games, from 7 to 15 (hard) [30]. Because VR balance games are not expected to influence cardiovascular capacity [35], we propose that other factors, such as the difficulty of the task for each individual, influenced the level of exertion experienced by participants. None of the participants needed or requested an extension of the 10-minute rest period during the study protocol.

Clinical reasoning for selecting patients after a stroke for whom serious games are meaningful is challenging, as the patient population is heterogeneous [5]. While we did not analyze subgroups in this study due to sample size limitations, we note that participants’ abilities influenced gameplay. Participants in our study with moderate balance deficits (Mini-BESTest [Mini Balance Evaluation Systems Test] 8‐13 points; n=4) frequently experienced difficulties while playing games on both balance boards, particularly in placing their feet correctly due to muscle shortening, foot deformities, and increased muscle tone. For participants with very mild balance deficits to normal balance [33] (Mini-BESTest 24‐27 points; n=8), the custom system games were often too easy or monotonous. Therefore, we speculated that for highly functioning patients after a stroke, noncustom games are likely more suitable. However, this is strictly an observation that requires further investigation with a larger sample size.

Taken together, the findings suggest that both custom and noncustom serious games have a role in balance training for persons after a stroke. Noncustom games promote greater movement amplitude and frequency toward the stroke-affected side. Custom games may promote greater adherence based on positive flow, enjoyment, and likability findings. There has been a tendency to dismiss noncustom games, particularly those that are game-paced. However, this study suggests they have a role in stimulating exercise intensity with movement toward the affected side. Game designers may also need to guard against constraining movement displacement.

While the findings of this study generalize to patients in the early-to-late chronic phase after a stroke with moderate-to-very mild balance deficits or normal balance (Mini-BESTest 8–27/28 points), the sample size did not allow for the interpretation of the effect of balance deficit severity on gameplay movement performance and user experience. Future studies may consider the relationship between balance ability and performance on serious games with a strong balance requirement. Movement performance should also be analyzed for games that encourage COP movement in the anteroposterior direction.

### Limitations of the Study

The findings of this study generalize to games that use the COP as the marker of movement. These game mechanics are shared by many other games that use some form of platform to track movement. However, it is unclear whether the findings would be similar in games that use total body tracking. Differences between the 2 VR systems used, such as sampling frequency, board dimensions, surface area, and material properties, could influence COP sensitivity and thereby affect gameplay mechanics independently of game customization. These factors were not isolated in this study, and their potential contribution cannot be fully excluded. It was not possible to perfectly match all the custom and noncustom games.

The analysis of video recordings was challenging, even though Kinovea software is a valid and reliable tool for measurements within an angle range of 90˚ to 45˚ [36], with accuracy for measuring linear positions and velocities from 30 Hz video within 9% [37]. The use of 3D motion analysis systems would increase the validity of the results. Another limitation of this study is the reduced number of participants available for movement analysis due to the concealed pelvic marker. We encountered several challenges due to compensatory pelvis rotations, which caused the marker to fall outside the appropriate plane or resulted in movements too small for measurement. Additionally, the marker was sometimes covered by the physiotherapist while providing physical guidance or ensuring safety. Consequently, for some recordings—particularly those of more demanding, faster games—we did not perform the analysis due to missing data.

### Conclusions

The findings of this study indicate that both customization and type of game pacing influence movement frequency, movement amplitude, enjoyment, flow state, and perceived exertion in VR balance games after a stroke. To our knowledge, this is the first study to parse the effects of game customization and type of game pacing on balance performance and the experience of patients after a stroke. Movement frequency and movement amplitude were significantly greater for all noncustom game–paced games and for half of the noncustom self-paced games. This is important to consider because weight-shifting toward the affected side is a common problem for patients after a stroke. Users’ flow state (including the sense of control and emotional experience subdomains) and likability (user preference) were significantly greater for custom game–paced games. There was no significant difference between custom and noncustom games for enjoyment. Therefore, it is important to consider both game customization (primarily for user experience, with custom games preferred) and type of game pacing (primarily for movement performance) when selecting serious games as interventions to improve balance for patients after a stroke. These findings contribute to the body of knowledge that guides clinicians’ clinical reasoning in selecting serious games for rehabilitation.

## Supplementary material

10.2196/88179 Multimedia Appendix 1 Noncustom system (Nintendo Wii Fit, Wii Balance Board, Nintendo Co.) and custom system (Equio, Kinestica d.o.o.) balance games description; comparison for self-paced and game-paced games.

## Abbreviations

COP: center of pressure
ES: effect size
FAC: Functional Ambulation Categories
FSSOT: Flow State Scale for Occupational Tasks
Mini-BESTest: Mini Balance Evaluation Systems Tests
mPACES: Modified Physical Activity Enjoyment Scale
RPE: Borg Rating of Perceived Exertion Scale
VR: virtual reality
