# Supplementary material for: Game Customization and Pace Effects on Movement Performance and the User Experience During Serious Games for Balance Among People After a Stroke: Cross-Sectional Repeated Measures Study
Source: JMIR Serious Games. 2026 Jun 17;14:e88179. doi: 10.2196/88179 (PMC13346641; doi:10.2196/88179)
Supplement: Multimedia Appendix 1 [file games-v14-e88179-s001.pdf]

Multimedia Appendix

Table S1. Noncustom system (Nintendo Wii Fit, Wii Balance Board, Nintendo Co.) and custom system (Equio, Kinestica d.o.o.) balance games description; comparison for self-paced and game-paced games.

SELF-PACED GAMES	
**WII**	**EQUIO**
**Penguin Slide**<br>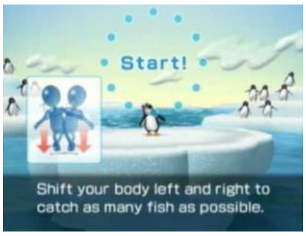<br><br>**Direction of movement:** M/L.<br>**Description:** Standing on the board, shift the body weight mediolaterally to tilt the ice platform on which the penguin avatar stands. The goal is to catch as many fish as possible without falling off the platform. There is no penalty for falling off.<br>**Scoring:** Based on the number and type of fish caught (blue fish – 1 point; green fish – 2 points; red fish – 10 points).<br>**Feedback:**<br>*Results:*<br>VF – display of time and points awarded for each fish caught.<br>AF – sound when a fish is caught.<br>*Performance:*<br>VF – the avatar's body moves according to the player's weight transfer.	**Lights**<br>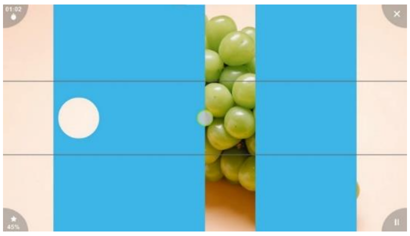<br>**Direction of movement:** M/L.<br>**Description:** Standing on the board, shift the body weight mediolaterally to direct the small grey circle (center of pressure) to the big circle on the screen and reveal part of the photograph. The aim of the game is to reveal the whole photograph. The player should not cross the two horizontal lines (A/P control).<br>**Scoring:** The percentage of success is calculated based on movement speed and accuracy. Stars are awarded.<br>**Feedback:**<br>*Results:*<br>VF – display of part of the photograph and the whole photograph at the end. Percentage to show the score.<br>AF – sound when the small circle touches the big one and when the photograph is revealed.<br>*Performance:*<br>VF – when the horizontal line is crossed the small circle turns red.
**Balance Bubble**<br>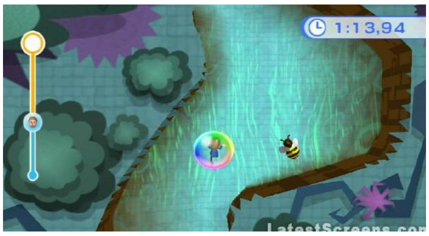<br><br>**Direction of movement:** all<br>**Description:** Standing on the board, shift the body weight in all directions to guide a bubble along a river. Avoid obstacles (walls, rocks, bee) to prevent the bubble from bursting. Speed increases with forward weight transfer. Game difficulty increases through water currents and visual distractions.<br>**Scoring:** Based on task duration and distance traveled.<br>**Feedback:**<br>*Results:*<br>VF – schematic display of the avatar's position on the course, time, and distance traveled.<br>*Performance:*<br>VF – the avatar's body moves according to the player's weight transfer.<br>AF – sound when the bubble approaches or hits a wall or rock.	**Labyrinth**<br>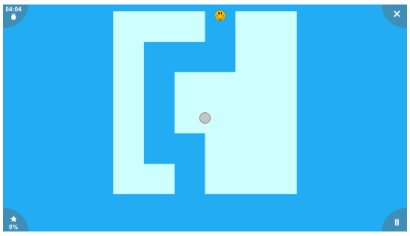<br>**Direction of movement:** A/P (simple version), all in advanced.<br>**Description:** Standing on the board, shift the body weight to move the grey circle (center of pressure) to the green circle that appears at the start. Then to follow the path of the labyrinth. The aim of the game is to guide the grey circle to the smiley face without touching the white edge.<br>**Scoring:** Percentage of success is calculated according to the path taken by the grey circle on the screen. The score is reduced if the grey circle goes outside the outlined path. Stars are rewarded.<br>**Feedback:**<br>*Results:*<br>VF – percentage of success to so show the score. When the grey circle touches the smiley, the maze disappears and a new one appears:<br>AF – sound signal when the grey circle touches the smiley.<br>*Performance:*<br>VF - the grey circle turns red when it moves outside the outlined path.<br>AF - sound when the grey circle moves outside the outlined path.

<table>
<tr><td>

<u>**Tilt Table**</u>

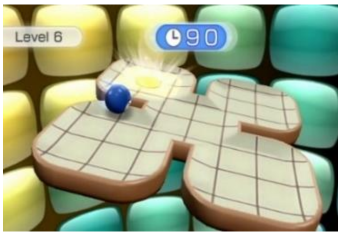

Direction of movement: all
Description: Standing on the board, shift the body weight in all directions to tilt the table on which balls are rolling. The objective is to guide the balls into the holes on the table. The table tilt corresponds directly to the player's weight transfer. When all balls are successfully guided into the holes, the player advances to the next level. Each level is time-limited; if the task is completed within the time limit, additional time is awarded for subsequent levels.
Scoring: 10 points are awarded for each successfully completed level.
Feedback:
Results:
VF – display of remaining time.
AF – sound when a ball falls into a hole.
Performance:
VF – tilt of the table on the screen corresponds to the player's weight transfer.

</td><td>

<u>**Tilt Table**</u>

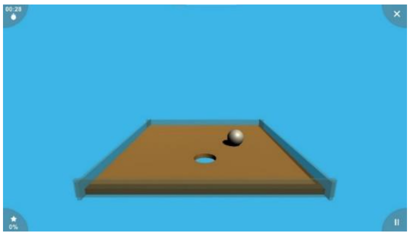

Direction of movement: all
Description: Standing on the board, shift the body weight in all directions to tilt the table on which one ball is rolling. The aim of the game is to roll the ball into the hole.
Scoring: Percentage of success is calculated depending on the path the ball takes on the table. The ideal path to the hole and the actual path taken by the ball are compared. If the ball falls off the table, the path is assumed to vary significantly.
Feedback:
Results:
VF – on a hit, the hole disappears, and a new hole appears.
AF – sound signal when the ball hits the hole or the edge.
Performance:
VF – tilt of the board in accordance with the player's weight transfer.

</td></tr>
<tr><td colspan="2">GAME-PACED GAMES</td></tr>
<tr><td>WII</td><td>EQUIO</td></tr>
<tr><td>

<u>**Soccer Heading**</u>

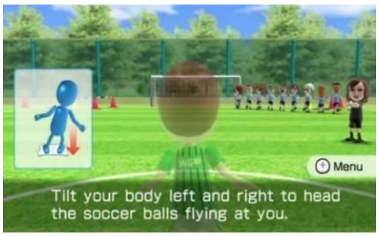

Direction of movement: M/L.
Description: Standing on the board, shift the body weight mediolaterally to control the avatar, who must head the ball into the goal. Avoid hitting other objects (e.g., shoe, panda).
Scoring: Based on the number of successfully headed balls, consecutive successful hits, and incorrectly hit objects.
Feedback
Results:
VF – display of points, remaining balls, and total gained and lost points.
AF – sound when the ball is successfully hit, missed, or an incorrect object is hit.
Performance:
VF – the avatar's body moves according to the player's weight transfer; on-screen text emphasizes weight shift between the lower limbs.

</td><td>

<u>**Block Breaker**</u>

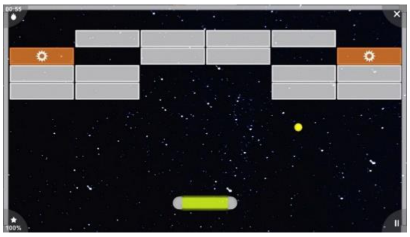

Direction of movement: M/L.
Description: Standing on the board, shift the body weight mediolaterally to control the plate and bounce the ball. The aim of the game is to hit all the blocks that are on top of the screen with the ball.
Scoring: The percentage of success is calculated based on the number of balls bounced and the number of balls missed. Stars are awarded.
Feedback
Results:
VF – when a ball hits the block, the block shatters.
AF – sound when the block is hit and when the ball bounces off the plate.
Performance:
VF – the plate on the screen moves according to the player's movement.

</td></tr>
</table>

Ski slalom

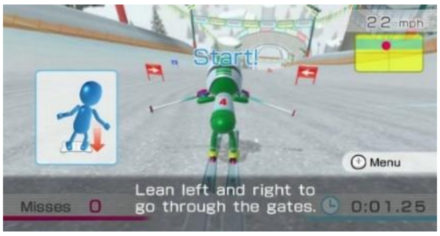

Direction of movement: M/L; A to increase speed
Description: Standing on the board, shift the weight mediolaterally to guide the avatar through 19 gates. The center of pressure is displayed on the screen. Game speed increases when the center of pressure (red dot) remains within the blue zone.
Scoring: Based on completion time and number of missed gates.
Feedback:
Results:
VF – display of avatar speed, time, and number of missed gates; wind effects indicate increased speed.
AF – sound when gates are successfully passed or missed.
Performance:
VF – the avatar's body moves according to the player's weight transfer; speed increases when the center of pressure is maintained within the blue zone.

Racer

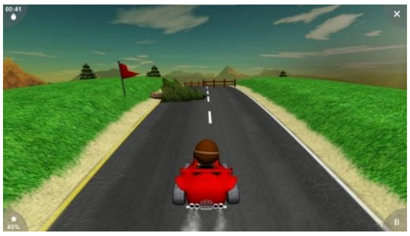

Direction of movement: M/L, also A at higher difficulties to increase speed.
Description: Standing on the board, shift the body weight mediolaterally to move the car to collect coins and avoid various obstacles. The speed increases as the weight is transferred forward.
Scoring: Percentage of success is calculated based on the number of obstacles avoided and coins collected. Stars are awarded.
Feedback:
Results:
VF - percentage of success to show the result.
AF - sound when an obstacle is hit, coin successfully collected, or ramp jumped.
Performance:
VF – the car on the screen moves in accordance with the player's weight transfer.

Snowboard Slalom

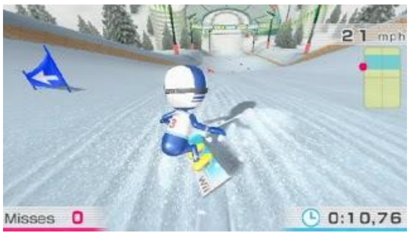

Direction of movement: A/P
Description: The board is rotated 90° clockwise. Standing on the board, the player faces left toward the screen and shifts the body weight anteroposteriorly to guide the avatar between turning markers. Speed increases when weight is transferred to the front (left) foot and the center of pressure (red dot) remains within the blue zone.
Scoring: Completion time plus a 7-second penalty for each error.
Feedback
Results:
VF – display of avatar speed, time, and number of missed turns; wind effects indicate increased speed.
AF – sound when turns are successfully passed or missed.
Performance:
VF – the avatar's body moves according to the player's weight transfer; speed increases when the center of pressure remains within the blue zone.

Shark

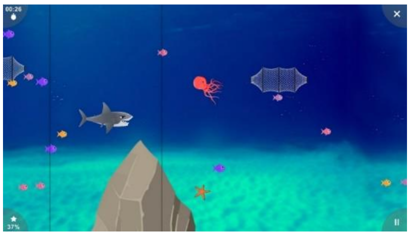

Direction of movement: A/P
Description: Standing on the board, shift the body weight anteroposteriorly to guide the shark around the obstacles (rocks, nets) and eat as much fish as possible. The player should not cross the two vertical lines (M/L control).
Scoring: Percentage of success is calculated based on the number and type of fish eaten. The score is lowered when the shark collides with obstacles and when the shark does not eat for a long time.
Feedback
Results:
VF – percentage of success to show the result. Shark flashes when hit by an obstacle; the shark is caught when hit by a net. A bubble appears when the shark eats a larger catch.
AF – sound when the shark catches a fish or hits an obstacle.
Performance:
VF – the shark in the game moves according to the player's weight transfer. When the shark crosses the boundary lines, they turn red.
AF – sound when the shark crosses the boundary lines for a longer period.

A, anterior direction; A/P, anteroposterior direction; M/L mediolateral direction; VF, visual feedback; AF auditory feedback; description of non-custom games refers to Deutsch et al. 2011.
